# Human galectin-1 and galectin-3 promote *Tropheryma whipplei* infection

**DOI:** 10.1080/19490976.2021.1884515

**Published:** 2021-02-12

**Authors:** Diyoly Ayona, Sandra Madariaga Zarza, Ludovic Landemarre, Benoît Roubinet, Philippe Decloquement, Didier Raoult, Pierre-Edouard Fournier, Benoit Desnues

**Affiliations:** a Aix Marseille Univ, IRD, APHM, MEPHI, Marseille, France; b IHU-Méditerranée Infection, Marseille, France; c Glycodiag, Rue De Chartres, BP6759, 45067, Orléans cedex 2, France; d Aix Marseille Univ, IRD, APHM, VITROME, Marseille, France

**Keywords:** Galectin-1, galectin-3, glycans, glycosylation, infection, *Tropheryma whipplei*, macrophage

## Abstract

*Tropheryma whipplei*, is an actinobacterium that causes different infections in humans, including Whipple’s disease. The bacterium infects and replicates in macrophages, leading to a Th2-biased immune response. Previous studies have shown that *T. whipplei* harbors complex surface glycoproteins with evidence of sialylation. However, the exact contribution of these glycoproteins for infection and survival remains obscure. To address this, we characterized the bacterial glycoprofile and evaluated the involvement of human β-galactoside-binding lectins, Galectin-1 (Gal-1) and Galectin-3 (Gal-3) which are highly expressed by macrophages as receptors for bacterial glycans.

*Tropheryma whipplei* glycoproteins harbor different sugars including glucose, mannose, fucose, β-galactose and sialic acid. Mass spectrometry identification revealed that these glycoproteins were membrane- and virulence-associated glycoproteins. Most of these glycoproteins are highly sialylated and N-glycosylated while some of them are rich in poly-N-acetyllactosamine (Poly-LAcNAc) and bind Gal-1 and Gal-3. *In vitro, T. whipplei* modulates the expression and cellular distribution of Gal-1 and Gal-3. Although both galectins promote *T. whipplei* infection by enhancing bacterial cell entry, only Gal-3 is required for optimal bacterial uptake. Finally, we found that serum levels of Gal-1 and Gal-3 were altered in patients with *T. whipplei* infections as compared to healthy individuals, suggesting that galectins are also involved *in vivo*.

Among *T. whipplei* membrane-associated proteins, poly-LacNAc rich-glycoproteins promote infection through interaction with galectins. *T. whipplei* modulates the expression of Gal-1 and Gal-3 both *in vitro* and *in vivo*. Drugs interfering with galectin–glycan interactions may provide new avenues for the treatment and diagnosis of *T. whipplei* infections.

## Introduction

*Tropheryma whipplei* is the causative agent of Whipple’s disease (WD),^1^ a rare systemic illness that mainly affects middle-aged Caucasian men.^2^ Classically, the disease occurs with severe infection of the small intestinal lining^3^ which is characterized by the infiltration in the lamina propria by heavily infected large foamy macrophages strongly stained by the Periodic acid Schiff (PAS) reagent.^4^ Later the infection may spread into different organs, mostly eyes, central nervous system and heart.^5,6^ WD can be fatal without early and proper treatment.^7^ The typical characteristics of the disease are weight loss, diarrhea, arthralgia, fever and hyper pigmentation of the skin^2^ and fat accumulation in the small intestine and mesenteric lymph nodes.^8^ *T. whipplei* infections can also manifest as chronic localized infections, such as endocarditis and encephalitis.^9,10^ Beside chronic infections, the bacteria can also cause acute infections mainly, gastroenteritis, pneumonia, and bacteremia.^5^ Most individuals develop a protective immune response against *T. whipplei* infection^11,12^ and asymptomatic carriage of *T. whipplei* is common.^13^ The development of the disease in a minor fraction of the population probably arise from a subtle genetic predisposition or an immune defect.^5^ Indeed, in patients, impaired bactericidal activity toward *T. whipplei* is associated with alternative activation of phagocytic cells and an anti-inflammatory response, suggesting a defect in macrophage functions during WD.^11,12,14^

The bacterium has a peculiar trilamellar membrane which is absent in other Gram positive bacteria.^2,4^ *T. whipplei* is the only known reduced genome (<1Mbp) bacteria in the class of Actinobacteria.^15^ Its membrane contains polysaccharides with N-linked glycosylation and sialylation making the bacteria positive for PAS staining.^16^ Among those membrane-associated glycoproteins, a WiSP (Whipplei Surface Proteins) protein called GpTw110 with an apparent molecular weight of 110 kDa has been identified.^16^ Electron microscopy images of *T. whipplei* cultured in HEL cells show that bacteria can form both intracellular and extracellular biofilms.^4^ Interestingly, the bacteria lose their glycosylation with prolonged culture, and this is associated with impaired replication in macrophages. In addition, GpTw110 is the main antigen recognized by immunoglobulins. Of note, serum samples from patients have a lower serologic response compared to asymptomatic carriers to *T. whipplei* and deglycosylated bacterial glycoproteins hinder the immune reactivity. Therefore, *T. whipplei* glycoproteins are believed to play an important role during bacterial replication and for immune evasion.^16^

Galectins are a family of carbohydrate-binding proteins (also known as S-type lectins) which are found in vertebrates, in some invertebrates but also in microorganisms. In humans, galectins have a broad spectrum of cellular and pathophysiological functions,^17,18^ primarily mediated by carbohydrate recognition/interaction.^19^ Members of this family are characterized by their distinct and evolutionary conserved carbohydrate recognition domain (CRD) which binds β-galactosides.^17^ Generally galectins have a higher affinity for complex N-glycans and poly-N-acetyllactosamine (poly-LacNAc). However, each member of the galectin family has a unique glycan affinity spectrum.^20^ Based on structural differences, galectins are categorized into the proto-, tandem-repeat and chimera-types. Proto- and chimera-type galectins have one CRD while tandem-repeat galectins have two distinct CRDs. Proto-type and tandem repeat galectins can non-covalently homodimerize and chimera-type form oligomers.^17^ In addition, mammalian galectins can be found both intracellularly in the cytosol or associated with cellular organelles, or extracellularly, remaining free soluble molecules or associated with cell membrane and extracellular matrices.^18,21^ Thereby, galectins bind not only intrinsic glycans but also extrinsic glycans (e.g. glycans on pathogens).^22^

Among different galectins, proto-type Gal-1 (14.5 kDa) and chimera-type Gal-3 (26 kDa) are highly expressed and secreted by immune cells, particularly by myeloid cells for which they are crucial for mediating immune cell migration, proliferation, adhesion and signaling.^23^ Hence, these two galectins are considered as key regulators of immune responses.^24–28^ During infections, Gal-1 and Gal-3 mediate immune responses primarily by interacting with glycans from pathogen and/or host glycans. Thereby, they can interfere with pathogen adherence and cell entry (e.g. Dengue virus),^29^ act as pattern recognition receptors (PRRs) exerting antimicrobicidal effects (e.g. *Candida* spp.),^30^ or inhibit microbial growth (e.g. *Streptococcus pneumonia*).^31^ However, some pathogens have evolved strategies to utilize host galectins to enhance host cell entry and/or to subvert immune responses.^32^ For example, Gal-1 enhances adherence of HIV-1 and *Chlamydia trachomatis* to their target cells by bridging glycans on host cell,^33,34^ while for *Trichomonas vaginalis*, Gal-1 not only enhances the adherence but also subverts immune responses in favor of the pathogen.^35,36^ Similarly, Gal-3 interaction with glycans expressed on *Trypanosoma cruzi* favors parasite attachment to target cells as well as immune evasion.^37^

The involvement of *T. whipplei* glycoproteins in infection is far from understood. Therefore, in this study we aimed at investigating the role of galectin–glycan interaction in *T. whipplei* infection. We found that both Gal-1 and Gal-3 bind poly-LacNAc-rich bacterial glycoproteins and enhance bacterial cell entry and that Gal-3 deficiency significantly reduces *T. whipplei* uptake by macrophages. Moreover, we found that sera from patients with *T. whipplei* infection have increased and decreased level of Gal-1 and -3, respectively, as compared with healthy controls. Thereby, we identified that *T. whipplei* utilizes its glycans to facilitate the infection via Gal-1- and Gal-3-mediated interaction.

## Results

### T. whipplei harbors variety of glycans and glycoproteins that bind Gal-1 and Gal-3 in a β-galactose dependent manner

**Figure 1.**
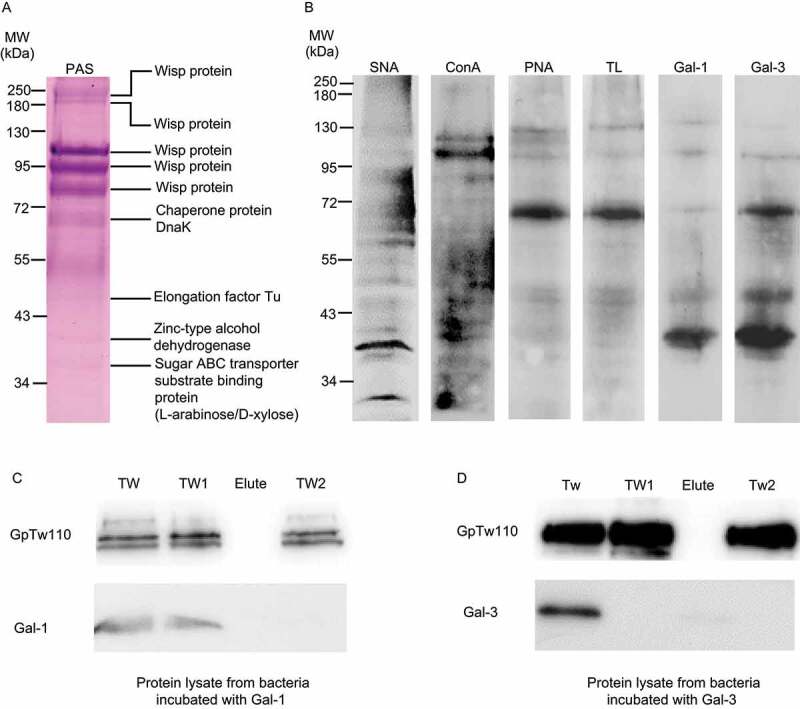
***T. whipplei harbors a variety of glycans and glycoproteins that bind Gal-1 and Gal-3 in a β-galactose-dependent manner.*** (a) Identified bacterial glycoproteins by mass spectrometry using PAS positive proteins bands from PAS-stained *T. whipplei* whole cell protein lysate resolved in SDS-PAGE. (b) Reactivity of SNA, ConA, PNA, TL, Gal-1 and Gal-3 with *T. whipplei* whole cell protein lysate resolved on SDS-PAGE and transferred on nitrocellulose. (c and d) Bacteria were incubated with recombinant Gal-1 (c) or Gal-3 (d) before washing twice with lactose. Bacterial pellets incubated with recombinant galectins (TW), bacterial pellets incubated with recombinant galectins and washed once with lactose (TW1), elutes from TW1 bacterial pellets, bacterial pellets incubated with recombinant galectins and washed consecutively twice with lactose (TW2) were subjected to immunoblotting to evaluate the displacement of bound galectin to bacteria. Results are representative of at least three independent experiments

In a first set of experiments, we performed PAS staining on bacterial whole cell lysate subjected to SDS/PAGE. As previously observed,^4^ we found that bacteria harbor several PAS-positive glycoproteins that we further identified by mass spectrometry as 5 WiSPs, chaperone protein DnaK (Heat-shock protein 70), elongation factor Tu (EF-Tu), zinc-type alcohol dehydrogenase and sugar ABC transporter substrate-binding protein (L-arabinose/D-xylose) (Figure 1a**, Table S1)**. We next characterized the glycoprofile of *T. whipplei* by analyzing its interaction with several lectins using two different methods, lectin microarray **(Fig. S1)** and lectin blotting (Figure 1b). Results indicated that *T. whipplei* glycoproteins harbor different types of glycans including glucose, mannose, fucose, N-acetylglucosamine (GlcNAc) and β-galactose. Bacterial whole-cell protein reactivity with different lectins indicated the presence of these glycans as follows: Concanavalin A (ConA) for α-mannose/α-glucose binding and N-glycans recognition (Figure 1b **and Fig. S1)**, *Burkholderia cenocepacia* lectin A (BC2LA) for mannose-specific glycans, *Pseudomonas aeruginosa* I lectin (PA-IL) and *Ulex europaeus* agglutinin I (UEA-I) for fucose glycans, *Griffonia simplicifolia* lectin II (GSL-II) for GlcNAc (**Fig. S1)**, peanut agglutinin (PNA) for β-galactose (Figure 1b **and Fig. S1)** and tomato lectin (TL) for poly-LacNAc and GlcNAc (Figure 1b). In addition, a strong reactivity was observed for *Trichosanthes japonica* agglutinin I (TJA-1) **(Fig. S1)** and *Sambucus nigra* agglutinin (SNA) lectins (Figure 1b **and Fig. S1)**, suggesting the presence of sialic acids moieties. Several bacterial proteins strongly interacted with SNA, including all PAS-stained glycoproteins as well as bands within the ranges of 95–55 kDa (Figure 1a, b). ConA strongly reacted with 4 major PAS-positive bands between 130 and 72 kDa and low molecular weight (LMW) bands below 55 kDa (Figure 1a). Blotting *T. whipplei* whole cell lysate with PNA and TL resulted in almost the same binding pattern, suggesting the presence of β-galactose as poly-LacNAcs (Figure 1a).

Finally, we found that both Gal-1 and Gal-3 bind at least 8 glycoproteins (Figure 1b), among which some corresponded to the glycoproteins that we identified by mass spectrometry, including the ~110 kDa WiSP (which represents the previously known as GpTw110), the chaperone protein DnaK, EF-Tu, zinc-type alcohol dehydrogenase and sugar ABC transporter substrate-binding protein (L-arabinose/D-xylose). However, Gal-3 appeared to have a higher reactivity than Gal-1 against the chaperone protein DnaK. These data suggest that *T. whipplei* harbors sialylated, N-glycosylated and poly-LacNAc-rich glycoproteins. The β-galactose-dependent binding of Gal-1 (Figure 1c) and Gal-3 (Figure 1d) was further confirmed by lactose competitive binding assay. We found that two lactose washes were required to completely remove bound Gal-1 from bacteria, while only one was necessary for Gal-3. In addition, we were able to detect released Gal-3 in the elute fraction from the first lactose washing (Figure 1d) but released Gal-1 from the first lactose washing was untraceable (Figure 1c). Hence, this result confirmed that Gal-1 and Gal-3 binding to *T. whipplei* depends on β-galactose.

### T. whipplei interacts with cellular Gal-1 and Gal-3 and modulates their expression and subcellular distribution in vitro

**Figure 2.**
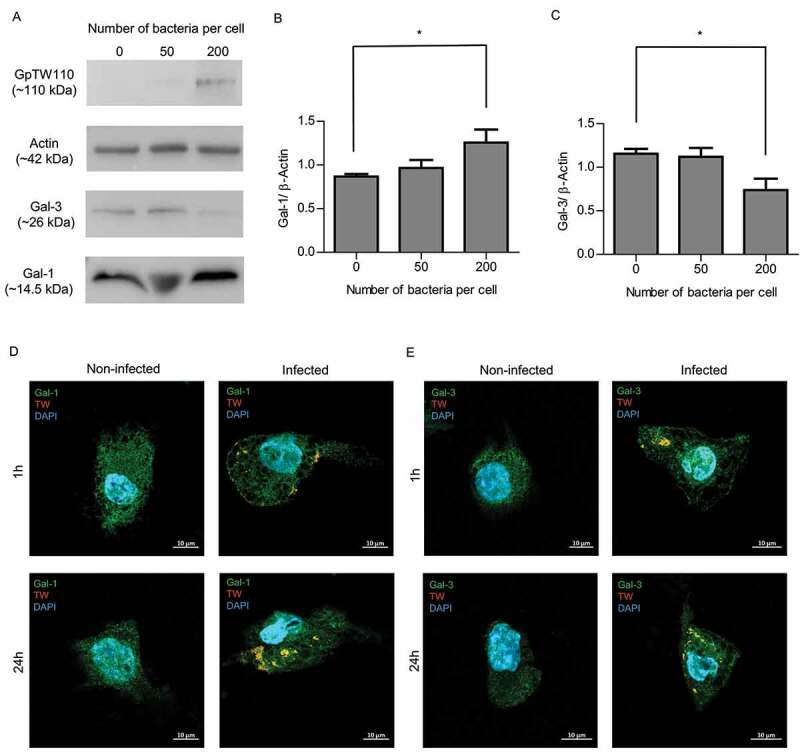
***T. whipplei interacts with cellular Gal-1 and Gal-3, and modulates their expression and subcellular distribution.*** (a) *T. whipplei*-specific GpTw110, actin, Gal-1 and Gal-3 immunoblotting of whole cell protein lysates obtained from macrophages exposed to 50 and 200 bacteria per cell for 24 h along with non-infected control. Band intensity were normalized to actin by densitometry analysis of Gal-1 (b) and Gal-3 (c). In B and C densitometry analysis represent the mean value of four independent experiments and bars represent the mean ± SEM. *P < .05, by unpaired-T test relative to the uninfected control. (d and e) Confocal microscopy of subcellular Gal-1 (d) and Gal-3 (e) distribution upon infection, respectively. Immunofluorescence images represent macrophages exposed or not to *T. whipplei* for 1 h (Top) and 24 h (Bottom). Gal-1 and Gal-3 were labeled with anti-rabbit Gal-1 and anti-rabbit-Gal-3, respectively, followed by a goat anti-rabbit Alexa488-labeled IgG (green). *T. whipplei* was detected with anti-*T. whipplei* antibody followed by anti-mouse Alexa555-labeled IgG (Orange) and DNA was stained with DAPI (Blue). Co-localization of bacteria with Gal-1/-3 is indicated by yellow color

Based on the role of Gal-1 and Gal-3 in immune response, we next aimed at defining the significance of *T. whipplei* interaction with these galectins. Macrophages were incubated with *T. whipplei* for 24 h and changes in Gal-1 and Gal-3 expression were assessed by immunoblotting. We found that when cells were infected with 200 bacteria per cell, expression of Gal-1 was increased and that of Gal-3 was decreased (Figure 2a). These changes in protein expression were further quantified by densitometry and were found significant compared to the non-infected control (Figure 2b, C). We next performed immunofluorescence staining to examine the interaction between *T. whipplei* and galectins. Confocal microscopy analysis of infected and non-infected cells demonstrated that *T. whipplei* drastically affect the cellular distribution of both Gal-1 and Gal-3 after 1 h or 24 h of infection (Figure 2d, E). Gal-1 and Gal-3 not only co-localized with bacteria but also accumulated at infected areas, disturbing the uniform cellular distribution. The Z-sections analysis of infected cells further confirmed the significant association of bacteria with membrane and cytosolic Gal-1 and Gal-3 and recruitment of Gal-1 **(See also Fig. S2 and Movie S1)** and Gal-3 to bacteria **(See also Fig. S3 and Movie S2)**. Overall, these data revealed that *T. whipplei* modulates Gal-1 and Gal-3 expression, binds Gal-1 and Gal-3 and alters their cellular distribution.

### Gal-1 and Gal-3 promote T. whipplei cell entry via galectin-glycan mediated interaction

**Figure 3.**
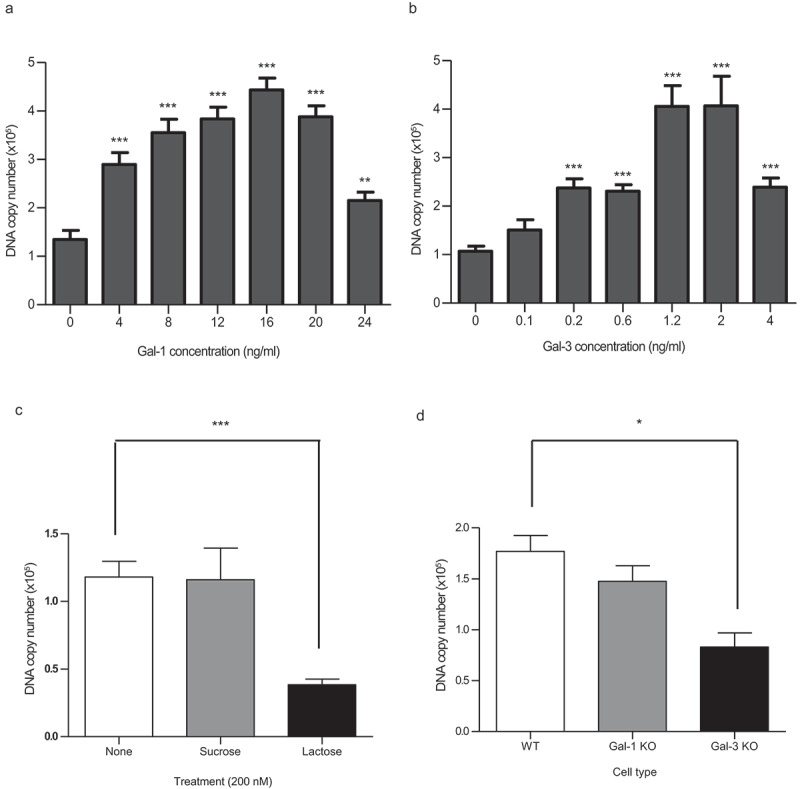
***Gal-1 and Gal-3 promote T. whipplei cell entry via galectin-glycan mediated interaction.*** (a and b) Bacterial cell entry in the presence of increasing concentration of recombinant Gal-1 (a) and Gal-3 (b) after 3 h of infection. Cell infection in the absence of added Gal-1 and Gal-3 was used as controls. (c) Bacterial cell entry after 2 h of infection in the presence of lactose or sucrose (200 mM) along with the control without any additive. (d) Bacterial cell entry after 3 h of infection in BMDMs from WT, Gal-1 KO and Gal-3 KO mice. In A, B, C, and D, *T. whipplei* DNA copy number was determined by qPCR using *T. whipplei*-specific primers. Bars represent the mean ± SEM of 4 independent experiments. In A and B, **P < .01 and ***P < .001 by unpaired T test relative to the control. In C and D, *P < .05 and ***P < .001 by Mann Whitney U test relative to the control

As galectins may be involved during bacterial internalization,^34,37^ we wondered whether extracellular recombinant soluble Gal-1 and Gal-3 would affect phagocytosis of *T. whipplei*. For this purpose, macrophages were infected in the presence of increasing concentrations of recombinant Gal-1 and Gal-3 for 3 h. We found that addition of both Gal-1 and Gal-3 significantly increase the intracellular bacterial load (Figure 3 A, B), till the cells reach a maximum cell infection, which was achieved with 16 ng/ml (Figure 3a) and 1.2–2 ng/ml (Figure 3b) of Gal-1 and Gal-3, respectively. The role of Gal-1 and Gal-3 in bacterial uptake was further confirmed by two different approaches. First, we used competitive experiments in which lactose was used as the competitive molecule for other ligands that bind galectins.^38^ Interestingly, we found that bacterial uptake was reduced by ~50% when cells were pre-incubated with 200 mM of lactose but not with 200 mM sucrose which was used as the negative control (Figure 3c). Second, we evaluated the bacterial uptake by Gal-1- and Gal-3-knock-out (KO) bone-marrow-derived macrophages (BMDMs). Our results indicated that bacterial uptake was significantly reduced in Gal-3 KO cells compared to both WT type and Gal-1 KO cells, although, Gal-1 KO cells also showed a slight decrease in bacterial uptake compared to WT cells, which was not significant (Figure 3d). Altogether, these results indicated that Gal-1 and/or Gal-3 interaction with bacterial glycans is critical for *T. whipplei* uptake by macrophages and that Gal-3 is required for efficient bacterial internalization.

### Circulating Gal-1 and Gal-3 levels are altered in patients with T. whipplei infections

**Figure 4.**
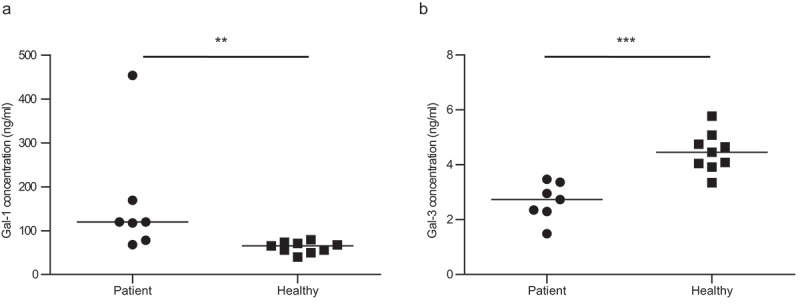
***Circulating Gal-1 and Gal-3 levels are altered in patients with T. whipplei infections.*** (a and b) Soluble Gal-1 (a) and Gal-3 (b) levels in sera from patients with *T. whipplei* infection and healthy individuals, determined by ELISA. **P < .005 and ***P < .001 by Mann-Whitney U test relative to the healthy control

Finally, we investigated Gal-1 and Gal-3 levels in sera from healthy individuals and patients with *T. whipplei* infections (Figure 4). We found that Gal-1 levels were significantly higher in patients than in healthy controls with a median value of 119.9 ng/ml (range 68.20–453.8 ng/ml) in patients versus 65.03 ng/ml (range 39.60–79.44 ng/ml) in controls (Figure 4a). In contrast, Gal-3 levels were significantly lower in patients than in healthy controls, with a median value of 2.73 ng/mL (range 1.49–3.48 ng/ml) in patients versus 4.46 ng/ml (range 3.35–5.78 ng/ml) in controls (Figure 4b). Overall, our results showed that in patients with *T. whipplei* infections, serum Gal-1 and Gal-3 levels are significantly different from those measured in healthy individuals.

## Discussion

Activities of galectins during infection are primarily mediated through binding to glycans from pathogen and/or host. Gal-1 and Gal-3 are the most abundant galectins expressed by immune cells. Emerging evidences emphasize that in some cases, these galectin-glycan interactions may benefit the pathogen by providing the ability to adhere, by enhancing uptake as well as by modulating immune effector functions.^32^ *T. whipplei* is an intracellular bacterium which replicates in alternatively activated macrophages. Although, it has been shown that *T. whipplei* glycoproteins are important for bacterial replication in macrophages, their contribution during the infection process is not well understood. In this report, we aimed at evaluating the interaction of Gal-1 and Gal-3 with *T. whipplei* glycoproteins and their involvement during *T. whipplei* infection.

By using lectin microarray and lectin blotting, we showed that *T. whipplei* harbors glucose, mannose, fucose, galactose and sialic acid, and expresses several poly-LacNAc-rich glycoproteins which bind both Gal-1 and Gal-3. Some of these glycoproteins were subsequently identified by mass spectrometry. As expected, the identified glycoproteins have previously been reported as membrane-associated in both Gram-positive and Gram-negative bacteria. For example, EF-Tu, which controls several virulence-associated functions, reaches cell surface to interact with host extracellular matrix components such as fibronectin to facilitate adhesion.^39^ Similarly, the chaperone protein DnaK (Heat-shock protein 70), which plays a critical role in overcoming stress responses is associated with or in close proximity to the membrane.^40,41^ Interestingly, *T. whipplei* DnaK seems to play a significant role. Indeed, *T. whipplei* up-regulates the expression of DnaK upon heat shock^42^ and patients with classical Whipple’s disease have reduced peripheral T-cell reactivity against DnaK compared to that of healthy controls.^43^ Finally, and not surprisingly, we also identified WiSP as membrane-associated glycoproteins that bind galectins. Although their role during *T. whipplei* infection has not been clearly characterized, it was shown that prolonged axenic growth was associated with reduced WiSP glycosylation and impaired intracellular replication in macrophages.^16^ Overall, our data showed that most of the glycoproteins that we identified bind both Gal-1 and Gal-3, suggesting that galectins may contribute to the pathogenicity of *T. whipplei* through the interaction with these membrane- and virulence-associated glycoproteins.

TL binding as well as Gal-1 and Gal-3 binding to these glycoproteins strongly suggest the presence of poly-LacNac. Indeed, both Gal-1 and Gal-3 preferentially bind poly-LacNAc-rich glycans, but the affinity of Gal-3 is higher than Gal-1. For example, the dissociation constant (K_d_) of Gal-1 and Gal-3 for LacNAc oligomers having repeating numbers of 5 are 39 μM and 0.19 μM, respectively.^20^ However, SNA lectin binding also suggests that, in the meantime, most of these glycoproteins are sialylated. Several studies have shown that α-2, 6-sialylation inhibits galectin binding, except Gal-3 which binds internal LacNAc residues.^44^ As a result, α-2, 6-sialylation of terminal LacNAc residues from *T. whipplei* glycoproteins may limit Gal-1 binding, but not that of Gal-3 and may explain the reduced band intensity when whole-cell lysates were probed with Gal-1 as compared with Gal-3. In addition, although Gal-1 and Gal-3 have a higher affinity for complex N-glycans (K_d_ <8 μM),^20^ we did not observe extensive binding of Gal-1 or Gal-3 to N-glycosylated proteins. This could also be a consequence of sialylation of glycoproteins. Overall when comparing the binding pattern of SNA and ConA, it seems that *T. whipplei* high molecular weight (HMW) proteins (>55 kDa) are associated with sialylation while LMW proteins are associated with N-glycosylation. Finally, we confirmed that binding of Gal-1 and Gal-3 to bacterial glycoproteins was dependent on β-galactose since lactose was able to completely displace bound galectin from bacteria. Nevertheless, two consecutive lactose washings were required for complete removal of bound Gal-1 from bacteria while one was sufficient for Gal-3. This observation may be the consequence of the stronger affinity of Gal-1 for poly-LacNAc chains while Gal-3 forms multiple low-affinity interactions with internal LacNAc residues.^44^

We next showed that *T. whipplei* affects Gal-1 and Gal-3 expression and also modulates sub-cellular distribution of Gal-1 and Gal-3. Indeed, we found that upon infection, Gal-1 expression was increased while that of Gal-3 was decreased. In addition, *T. whipplei* disturbs the homogeneous distribution of Gal-1 and Gal-3 in macrophages and both Gal-1 and Gal-3 accumulate at infected areas of the cell where they co-localize with *T. whipplei*. Similarly, during *C. trachomatis* infection of HeLa cells the expression of Gal-1 is increased and Gal-1 accumulates nearby bacteria disturbing the homogenous cellular distribution of Gal-1.^34^ Such results convince the ability of pathogens to modulate galectin expression as well as the ability of galectins to act as PRRs for pathogens.

Several studies have demonstrated that external addition of soluble Gal-1 and Gal-3 either enhance or inhibit cell entry of some pathogens.^32^ For example, soluble Gal-1 enhances *C. trachomatis* uptake in a dose-dependent manner *in vitro*^34^ while for *T. cruzi*, cell adhesion is increased with the addition of soluble Gal-3.^37^ In contrast, addition of Gal-1 inhibits the internalization of Dengue virus and Nipah Virus.^29,45,46^ We demonstrated that both Gal-1 and Gal-3 enhance *T. whipplei* phagocytosis by macrophages in a dose-dependent manner. Interestingly, addition of recombinant human Gal-3 as low as 0.2 ng/ml was sufficient to double the uptake of *T. whipplei* while with recombinant human Gal-1, this was achieved with 4 ng/ml. In line with this, inhibition of galectin binding by lactose drastically decreased bacterial phagocytosis. Even though both soluble Gal-1 and Gal-3 enhance the phagocytosis of *T. whipplei* by macrophages, the difference in bacterial uptake by Gal-1 KO and Gal-3 KO cells revealed that only Gal-3 is critical for optimal bacterial uptake. Therefore, our results suggest that both galectins are important for bacterial adherence through glycan-galectin-mediated interaction and that Gal-3 is required for cell entry .

Different studies have addressed the regulation of Gal-1 and Gal-3 levels in sera from rheumatoid arthritis (RA) patients. According to these studies, RA patients have higher Gal-1 as well as Gal-3 levels in sera compared to healthy individuals.^47–49^ *T. whipplei* infections are commonly associated with joint manifestations mimicking seronegative RA.^50,51^ We found that expression of Gal-1 and Gal-3 was significantly higher and lower, respectively, in sera from patients with *T. whipplei* infection, as compared with healthy controls. Interestingly, in our study, 5 patients had symptoms of arthritis and the highest Gal-1 levels were measured from a patient with severe peripheral arthralgia. Therefore, further studies on larger sample size are worthwhile to understand whether the changes in serum Gal-1 and Gal-3 upon *T. whipplei* infection are associated with severity of arthritis and arthralgia.

Moreover, we found that *in vitro, T. whipplei* increased the expression of Gal-1 by macrophages while that of Gal-3 was reduced. According to several different studies, it seems that Gal-1 is more associated with anti-inflammatory functions^52–54^ while Gal-3 is mainly associated with pro-inflammatory functions.^55,56^ Both Gal-1 and Gal-3 are critical determinants of macrophage polarization. However, their exact contribution for activation of different macrophage phenotypes is still controversial.^57–61^ Interestingly, *T. whipplei* infection is associated with Th2-biased immune responses, including alternative activation/M2 polarization of macrophages.^11,14^ Compared to Gal-3, Gal-1 expression is higher after *in vitro* infection but also in the serum from patients. In addition, the optimal soluble Gal-1 concentration that increases phagocytosis *in vitro* is also higher than that of Gal-3. However, we found that only Gal-3 is critical as a cell entry mediator/receptor. It is therefore possible that at the beginning of the infection, both Gal-1 and Gal-3 enhance cell entry, and at later stages, optimal Gal-1 and Gal-3 levels are reached both locally and systemically to shape the *T. whipplei*-favorable Th-2 dominant microenvironment, although other factors are probably involved. Hence, circulating Gal-1 and Gal-3 levels may thus provide a snapshot of the polarization status of the immune response against *T. whipplei* infection.

Finally, several single nucleotide polymorphisms (SNPs) of Gal-1 and Gal-3 have been associated with increased or decreased Gal-1 and Gal-3 expression in sera.^62,63^ Such SNPs may also affect the uptake of the pathogen. Indeed, during enterovirus EV71 infection, a non-synonymous Gal-3 genetic variant is associated with lower virus titer than the wild-type allele.^62^ Similarly, it has been reported that susceptibility to influenza A (H7N9) infection is associated with functional variants of Gal-1 which affect its expression.^63^ When considering the opsonin effect of both Gal-1 and Gal-3 during *T. whipplei* infection and the spectrum of *T. whipplei* infections, it is worthwhile to assess the association between the type of the infection (carriage, chronic isolated or chronic systemic) and the polymorphism of Gal-1 and Gal-3. Finally, the drugs which are used to treat *T. whipplei* infection, doxycycline and hydroxychloroquine have been identified as chemical modulators of Gal-1 and Gal-3 expression in humans and their role against infection (e.g. Coronaviruses) or cancer may also arise from galectin inhibition.^64–67^

In conclusion, our study demonstrates that both Gal-1 and Gal-3 favor *T. whipplei* infection by enhancing the internalization of bacteria through glycan-galectin-mediated interaction, but only Gal-3 is required for optimal bacterial uptake. Bacteria also modulate the expression and distribution of Gal-1 and Gal-3. In addition, the enhanced Gal-1 and decreased Gal-3 levels in patients with *T. whipplei* infection may reflect galectin-mediated immune evasion by *T. whipplei*. Further studies are required to determine the involvement of specific *T. whipplei* glycoproteins in galectin recruitment and immune evasion. Therefore, the understanding of galectin–glycan interaction and the interplay of different galectins during *T. whipplei* infections may be of critical importance for the development of novel therapeutic and diagnostic approaches.

## Materials and methods

### Bacteria, cell culture and infection

*Tropheryma whipplei* str. Twist was cultured in axenic medium as previously described.^68^ THP-1 (ATCC® TIB-202) cells were obtained from the American Type Culture Collection. Cells were maintained in RPMI 1640 medium (Gibco) supplemented with 10% fetal bovine serum (FBS, Gibco) and 1% penicillin/streptomycin (Gibco) at 37°C and 5% CO_2_. Cells were kept at a minimum density of 3 × 10^5^ cells/ml and were passaged when reaching 8 × 10^5^ cells/ml. Cells were sub-cultured in 24 well plates or 6 well plates at a cell density of 5 × 10^5^ and 1 × 10^6^ cells/well, respectively. Phorbol-12-myristate 13-acetate (PMA) (Sigma-Aldrich) was added to a final concentration of 100 nM to differentiate THP-1 cells into macrophages. After 3 days, cells were gently washed once with 1X phosphate-buffered saline (PBS, Gibco) and incubated for an additional 24 hours in 10% FBS in RPMI 1640. To evaluate the bacterial cell entry in the presence of added recombinant Gal-1 and recombinant Gal-3, cells were infected in the presence of indicated recombinant Gal-1 or recombinant Gal-3 and incubated for 3 hours. To evaluate the impact of blocking galectin on bacterial cell entry, macrophages were pre-incubated 2 hours with and without 200 mM lactose or 200 mM sucrose and thereafter, cells were infected for 2 hours. To evaluate the Gal-1 and Gal-3 expression and cellular distribution cells were infected for 1 hour or 24 hours. Bone marrow-derived macrophages (BMDMs) from Gal-1 KO (kindly given by Dr. Stéphane Mancini, Cancer Research Center of Marseille, France), Gal-3 KO (kindly given by Dr. Ludger Johannes, Institut Curie, France) and C57BL/6 wild-type (WT, Charles River laboratories) mice were generated as previously described.^69^ Unless notified, macrophages were infected with 50 bacteria per cell and incubated 37°C and 5% CO_2._ At the end of each infection period, cells were washed twice with PBS to remove free bacteria.

### PAS staining

Bacteria (1 × 10^9^) were sonicated for 2 minutes at 20 W in TS-lysis buffer (7 M urea, 2 M Thiourea, 1% CHAPS). Protein quantity of bacterial cell lysate was determined by Bradford Assay (Bio-Rad). Amount of 40 µg from bacterial whole cell lysate was resolved on 10% SDS/PAGE. After migration, gel was incubated in 12.5% tri-chloro acetic acid for 30 minutes, rinsed with distilled water for 15 minutes and incubated for 50 minutes in 1% Periodic acid in 3% acetic acid. Excess periodic acid was removed by several washings with distilled water and the gel was incubated in distilled water at room temperature overnight. Schiff’s reagent (Sigma-Aldrich) was then added in the dark for 50 minutes and subsequently washed the gel with 0.5% metabisulfite for 10 minutes, trice. After removing excess stain by several washings with distilled water, the gel was reserved in 5% acetic acid and scanned.

### Trypsin digestion and identification of PAS-stained protein bands

Protein bands were cut from PAS-stained gel. Separated bands were washed several times with acetonitrile and water and digested overnight at room temperature into peptides using in-gel digestion with proteomics grade trypsin (Agilent Technologies). The peptides were extracted from the gel using acetonitrile. A matrix-assisted laser desorption/ionization time-of-flight spectrometry (MALDI-TOF-MS) on a Bruker Autoflex Speed spectrometer (Bruker Daltonics, Wissembourg, France) was used to identify the protein bands. The mass spectrometer was calibrated externally using Bovine serum albumin tryptic peptides. Peptide mixture (1 μL) was co-crystallized onto the anchorchip MALDI-TOF target plate with an equal amount of matrix solution (0.3 mg/mL of α-cyano-4-hydroxycinnamic acid in acetone and ethanol in 1:2 volume ratio and 0.1% trifluoroacetic acid). Mascot software was used for protein identification using peptide mass fingerprinting. Searches were performed against all available sequences in public databases.

### Lectin binding assays

Equal amounts of bacterial whole-cell lysate were resolved in 10% SDS/PAGE gel and transferred onto 0.45-µm nitrocellulose membranes (Bio-Rad). Membranes were incubated in carbo-free blocking buffer (Vector) for 1 h. For lectin-blotting, strips were probed with the following biotinylated lectins (5 µg/ml) (Vectors) for 1 hour: *Sambucus nigra* (SNA), *Lycopersicon esculentum* (Tomato) lectin (TL), Peanut agglutinin (PNA) and Concanavalin A (ConA). For blotting with Gal-1 and Gal-3, strips were incubated for 2 hours with biotinylated recombinant Gal-1 and Gal-3 (1:1000) (Peprotech). Thereafter, strips were washed with 0.3% Tween-20 in PBS and incubated with streptavidin-HRP (1:10000) (Sigma-Aldrich) for 1 hour followed by washings with 0.3% Tween-20 in PBS. Finally, strips were treated with ECL Western blotting substrate (Promega) and visualized under Fusion FX (Vilber Lourmat).

### Carbohydrate-dependent galectin binding of Gal-1 and Gal-3 to T. whipplei

Gal-1 and Gal-3 binding assay was performed as described previously.^34^ Briefly, bacteria were incubated with recombinant Gal-1 or Gal-3 (0.3 µg/ml) for 1 hour at 4°C, washed with PBS to remove unbound galectin and washed with lactose twice (500 μl, 200 mM) to remove bound galectin to bacteria. Equal amount of bacterial protein lysates obtained from bacterial pellets incubated with recombinant galectins (TW), bacterial pellets incubated with recombinant galectins and washed once with lactose (TW1), elutes from TW1 bacterial pellets, bacterial pellets incubated with recombinant galectins and washed consecutively twice with lactose (TW2) were resolved in 15% SDS/PAGE gel and transferred onto 0.45-µm nitrocellulose membrane. Membrane was blocked in carbo-free blocking buffer for 1 hour and incubated overnight at 4°C with anti-Gal-1 (1:1,000) or anti-Gal-3 (1:1,000) followed by incubation with goat anti-rabbit HRP-conjugated IgG (1:5000) (Invitrogen) for 1 hour. The *T. whipplei*-specific GpTw110 protein was used as loading control and labeled by incubating blots overnight at 4°C with GpTw110 specific anti-mouse polyclonal antibody (1:1000).^16^ Blots were visualized using ECL Western blotting substrate under Fusion FX (Vilber Lourmat).

### Immunoblotting

Cells were lysed with TS-lysis buffer and protein quantity was assessed by Bradford assay (Bio-Rad). Equal amount of protein lysates were loaded in 15% SDS/PAGE gel and proteins were transferred onto 0.45-µm nitrocellulose membranes. Membranes were incubated in carbo-free blocking buffer for 1 hour. To label Gal-1 and Gal-3 blots were incubated overnight at 4°C, respectively, with anti-Gal-1 (1:1,000) antibody and anti-Gal-3 (1:1,000) antibody followed by incubation with goat anti-rabbit HRP-conjugated IgG (1:5000) for 1 hour. *T. whipplei*-specific GpTw110 was used as the loading control for bacteria. Monoclonal Anti-β-Actin−Peroxidase antibody (1:25000) (Sigma-Aldrich) for 1 hour was used to label β-actin. After each incubation blots were washed in 0.3% Tween-20 PBS. Bands were visualized using ECL Western blotting substrate under Fusion FX (Vilber Lourmat).

### Immunofluorescence assay

Cells grown on coverslips were fixed in 3% paraformaldehyde for 15 minutes and permeabilized with Triton X-100 (0.1%) for 3 minutes. Anti-human Gal-1, anti-human Gal-3 (1:1000) and *T. whipplei*-specific mouse antibodies (1:2000) prepared as previously described (Liang, Z., 2002) were used to label Gal-1, Gal-3 and bacteria, respectively. Donkey Anti-Rabbit IgG (1:1000) (Alexa Fluor® 488, Invitrogen) and goat anti-mouse IGg (1:1000) (Alexa Fluor® 555, Invitrogen) were used as secondary antibodies for 45 minutes to label the two galectins and bacteria, respectively. Nuclei were labeled by with 4′,6-diamidino-2-phenylindole (1:500) for 15 minutes (DAPI, Invitrogen). After each labeling coverslips were washed several times with 5% FBS in PBS. Air-dried coverslips were mounted with mowiol and slides were observed using LSM 8000 Airyscan confocal microscope (Zeiss LSM 800) under oil immersion objective (63 × 1.5).

### DNA extraction and quantification of intracellular bacteria

Cells were lysed with Triton X-100 (1% in PBS) and DNA was extracted using QIAamp DNA MiniKit (Qiagen). The quantity and the quality of extracted DNA was assessed by nanodrop spectrophotometer (Nanodrop technologies). Quantitative polymerase chain reaction (qPCR) was performed using specific primers for *T. whipplei* 16S-23S ribosomal intergenic spacer region as described previously^4^ and Smart SYBR Green kit (Roche).Threshold cycles (Ct) were obtained using CFX Touch Real-Time PCR detection system (Bio-Rad). For each qPCR run, a standard curve was generated using a serial dilution ranging from 10^2^ to 10^8^ copies of the intergenic spacer region of *T. whipplei*.

### Immunoassays

A total of 7 patients with proven diagnosis of *T. whipplei* infection by a strategy developed in our institute^70^ were included in this study: 7 Whipple’s disease patients (6 Males; mean age 68 ± 10 years and 1 Female; age 65 years).The study was validated by the ethics of the Mediterranee Infection Institute under reference 2016–025. Serum samples from 9 healthy donors (5 Males; mean age 41.6 ± 9.4 years and 4 females; mean age 39.8 ± 2 years) were obtained from the Etablissement Français du Sang (Marseille, France) according the convention n°7828. Serum concentrations of Gal-1 and Gal-3 were determined by enzyme-linked immunosorbent assay (ELISA) according to the manufacturer’s instructions using immunoassays for Gal-1 from RayBiotech, Inc (Assay sensitivity 4.10 ng/mL) and for Gal-3 from Peprotech (Assay sensitivity <10 pg/mL).

### Quantification and statistical analysis

Densitometry analysis of Western blots was performed using Image J 1.52a software. Statistical Analysis was performed using GraphPad Prism 6.0 Software. Data represent the mean ± SEM of N experiments. For simple unpaired analysis between two independent groups, unpaired T test was used. Mann–Whitney U test was used when the dependent variable is not normally distributed. *P* values less than 0.05 were considered as statistically significant.

## Supplementary Material

Supplemental Material

## References

[1] Relman DA, Schmidt TM, MacDermott RP, Falkow S. Identification of the uncultured bacillus of Whipple’s disease. N Engl J Med. 1992;327:293–15. PMID: 1377787. doi:10.1056/NEJM199207303270501. 1377787 PMC13545976

[2] Dobbins WO III. The diagnosis of Whipple’s disease. N Engl J Med. 1995;332:390–392. PMID: 7529893. doi:10.1056/NEJM199502093320611. 7529893

[3] Ribaux C, Saguem MH, Allaz AF, Kovacsovics T. Whipple’s disease. Involvement of the small intestine with submucosal presentation. Ann Pathol. 1990;10:191–193. PMID: 1696824. 1696824

[4] La Scola B, Fenollar F, Fournier P-E, Altwegg M, Mallet M-N, Raoult D. Description of *Tropheryma whipplei* gen. nov., sp. nov., the Whipple’s disease bacillus. Int J Syst Evol Microbiol. 2001;51:1471–1479. PMID: 11491348. doi:10.1099/00207713-51-4-1471. 11491348

[5] Dolmans RA, Boel CE, Lacle MM, Kusters JG. Clinical manifestations, treatment, and diagnosis of *Tropheryma whipplei* infections. Clin Microbiol Rev. 2017;30:529–555. PMID: 28298472. doi:10.1128/CMR.00033-16.. 28298472 PMC5355640

[6] Marth T, Moos V, Müller C, Biagi F, Schneider T. *Tropheryma whipplei* infection and Whipple’s disease. Lancet Infect Dis. 2016;16:e13–e22. PMID: 26856775. doi:10.1016/S1473-3099(15)00537-X. 26856775

[7] Săraci G, Zaharie T, Zaharie GC, Vesa ŞC, Vişovan II, Gheban D, Pobîrci LO. Fatal Whipple’s disease with severe mental manifestations on relapse - case report and brief advances update. Rom J Morphol Embryol. 2019;60:319–323. PMID: 31263862. 31263862

[8] Whipple GH. A hitherto undescribed disease characterized anatomically by deposits of fat and fatty acids in the intestinal and mesenteric lymphatic tissues. Bull Johns Hopkins Hosp. 1907;18:382–393.

[9] Fenollar F, Célard M, Lagier JC, Lepidi H, Fournier P-E, Raoult D. *Tropheryma whipplei* endocarditis. Emerg Infect Dis. 2013;19:1721–1730. PMID: 24207100. doi:10.3201/eid1911.121356. 24207100 PMC3837638

[10] Compain C, Sacre K, Puéchal X, Klein I, Vital-Durand D, Houeto J-L, De Broucker T, Raoult D, Papo T. Central nervous system involvement in Whipple Disease: clinical Study of 18 patients and long-term follow-up. Medicine. 2013;92:324–330. PMID: 24145700. doi:10.1097/MD.0000000000000010. 24145700 PMC4553994

[11] Marth T, Kleen N, Stallmach A, Ring S, Aziz S, Schmidt C, Strober W, Zeitz M, Schneider T. Dysregulated peripheral and mucosal Th1/Th2 response in Whipple’s disease. Gastroenterology. 2002;123:1468–1477. PMID: 12404221. doi:10.1053/gast.2002.36583. 12404221

[12] Moos V, Schmidt C, Geelhaar A, Kunkel D, Allers K, Schinnerling K, Loddenkemper C, Fenollar F, Moter A, Raoult D. Impaired immune functions of monocytes and macrophages in Whipple’s disease. Gastroenterology. 2010;138:210–220. PMID: 19664628. doi:10.1053/j.gastro.2009.07.066. 19664628

[13] García-Álvarez L, Pérez-Matute P, Blanco JR, Ibarra V, Oteo JA. High prevalence of asymptomatic carriers of *Tropheryma whipplei* in different populations from the North of Spain. Enferm Infecc Microbiol Clin. 2016;34:340–345. PMID: 26585816. doi:10.1016/j.eimc.2015.09.006. 26585816

[14] Desnues B, Lepidi H, Raoult D, Mege J-L. Whipple disease: intestinal infiltrating cells exhibit a transcriptional pattern of M2/alternatively activated macrophages. J Infect Dis. 2005;192:1642–1646. PMID: 16206080. doi:10.1086/491745. 16206080

[15] Raoult D, Ogata H, Audic S, Robert C, Suhre K, Drancourt M, Claverie J-M. *Tropheryma whipplei* Twist: a human pathogenic Actinobacteria with a reduced genome. Genome Res. 2003;13:1800–1809. PMID: 12902375. doi:10.1101/gr.1474603. 12902375 PMC403771

[16] Bonhomme CJ, Renesto P, Desnues B, Ghigo E, Lepidi H, Fourquet P, Fenollar F, Henrissat B, Mege J-L, Raoult D. *Tropheryma whipplei* glycosylation in the pathophysiologic profile of Whipple’s disease. J Infect Dis. 2009;199:1043–1052. PMID: 19222368. doi:10.1086/597277. 19222368

[17] Cummings RD, Liu F-T, Vasta GR. Galectins. In: Varki A, Cummings RD, Esko JD, Stanley P, Hart GW, Aebi M, Darvill AG, Kinoshita T, Packer NH, Prestegard JH, et al., editors. Essentials of Glycobiology. 3rd ed. Cold Spring Harbor Laboratory Press (NY): Cold Spring Harbor; 2015. p. 469–480.

[18] Leffler H. Galectins structure and function–a synopsis. Results Probl Cell Differ. 2001;33:57–83. PMID: 11190679. doi:10.1007/978-3-540-46410-5_4. 11190679

[19] Kamili NA, Arthur CM, Gerner-Smidt C, Tafesse E, Blenda A, Dias-Baruffi M, Stowell SR. Key regulators of galectin-glycan interactions. Proteomics. 2016;16:3111–3125. PMID: 27582340; PMCID: PMC5832938. doi:10.1002/pmic.201600116. 27582340 PMC5832938

[20] Hirabayashi J, Hashidate T, Arata Y, Nishi N, Nakamura T, Hirashima M, Urashima T, Oka T, Futai M, Muller WE, et al. Oligosaccharide specificity of galectins: a search by frontal affinity chromatography. Biochim Biophys Acta. 2002;1572:232–254. PMID: 12223272. doi:10.1016/s0304-4165(02)00311-2. 12223272

[21] Johannes L, Jacob R, Leffler H. Galectins at a glance. J Cell Sci. 2018;131:jcs208884. PMID: 29717004. doi:10.1242/jcs.208884. 29717004

[22] Vasta GR. Galectins as pattern recognition receptors: structure, function, and evolution. Adv Exp Med Biol. 2012;946:21–36. PMID: 21948360; PMCID: PMC3429938. doi:10.1007/978-1-4614-0106-3_2. 21948360 PMC3429938

[23] Liu FT. Regulatory roles of galectins in the immune response. Int Arch Allergy Immunol. 2005;136:385–400. PMID: 15775687. doi:10.1159/000084545. 15775687

[24] Sundblad V, Morosi LG, Geffner JR, Rabinovich GA. Galectin-1: a Jack-of-All-Trades in the Resolution of Acute and Chronic Inflammation. J Immunol. 2017;199:3721–3730. PMID: 29158348. doi:10.4049/jimmunol.1701172. 29158348

[25] Camby I, Le Mercier M, Lefranc F, Kiss R. Galectin-1: a small protein with major functions. Glycobiology. 2006;16:137R–157R. PMID: 16840800. doi:10.1093/glycob/cwl025. 16840800

[26] Díaz-Alvarez L, Ortega E. The Many Roles of Galectin-3, a Multifaceted Molecule, in Innate Immune Responses against Pathogens. Mediators Inflamm. 2017;2017:9247574. PMID: 28607536. doi:10.1155/2017/9247574. 28607536 PMC5457773

[27] Rabinovich GA, Liu FT, Hirashima M, Anderson A. An emerging role for galectins in tuning the immune response: lessons from experimental models of inflammatory disease, autoimmunity and cancer. Scand J Immunol. 2007;66:143–158. PMID: 17635792. doi:10.1111/j.1365-3083.2007.01986.x. 17635792

[28] Blidner AG, Méndez-Huergo SP, Cagnoni AJ, Rabinovich GA. Re-wiring regulatory cell networks in immunity by galectin-glycan interactions. FEBS Lett. 2015;589:3407–3418. PMID: 26352298. doi:10.1016/j.febslet.2015.08.037. 26352298

[29] Toledo KA, Fermino ML, Andrade Cdel C, Riul TB, Alves RT, Muller VD, Russo RR, Stowell SR, Cummings RD, Aquino VH, et al. Galectin-1 exerts inhibitory effects during DENV-1 infection. PLoS One. 2014;9(11):e112474. PMID: 25392933. doi:10.1371/journal.pone.0112474. 25392933 PMC4231055

[30] Kohatsu L, Hsu DK, Jegalian AG, Liu FT, Baum LG. Galectin-3 induces death of Candida species expressing specific beta-1,2-linked mannans. J Immunol. 2006;177:4718–4726. PMID: 16982911. doi:10.4049/jimmunol.177.7.4718. 16982911

[31] Farnworth SL, Henderson NC, Mackinnon AC, Atkinson KM, Wilkinson T, Dhaliwal K, Hayashi K, Simpson AJ, Rossi AG, Haslett C, et al. Galectin-3 reduces the severity of pneumococcal pneumonia by augmenting neutrophil function. Am J Pathol. 2008;172(2):395–405. PMID: 18202191. doi:10.2353/ajpath.2008.070870. 18202191 PMC2312371

[32] Ayona D, Fournier PE, Henrissat B, Desnues B. Utilization of Galectins by Pathogens for Infection. Front Immunol. 2020;11:1877. PMID: 32973776. doi:10.3389/fimmu.2020.01877. 32973776 PMC7466766

[33] St-Pierre C, Manya H, Ouellet M, Clark GF, Endo T, Tremblay MJ, Sato S. Host-soluble galectin-1 promotes HIV-1 replication through a direct interaction with glycans of viral gp120 and host CD4. J Virol. 2011;85:11742–11751. PMID: 21880749. doi:10.1128/JVI.05351-11. 21880749 PMC3209312

[34] Lujan AL, Croci DO, Gambarte Tudela JA, Losinno AD, Cagnoni AJ, Mariño KV, Damiani MT, Rabinovich GA. Glycosylation-dependent galectin-receptor interactions promote *Chlamydia trachomatis* infection. Proc Natl Acad Sci U S A. 2018;115:E6000–E6009. PMID: 29891717. doi:10.1073/pnas.1802188115. 29891717 PMC6042088

[35] Bastida-Corcuera FD, Okumura CY, Colocoussi A, Johnson PJ. *Trichomonas vaginalis* lipophosphoglycan mutants have reduced adherence and cytotoxicity to human ectocervical cells. Eukaryot Cell. 2005;4:1951–1958. PMID: 16278462. doi:10.1128/EC.4.11.1951-1958.2005. 16278462 PMC1287856

[36] Okumura CY, Baum LG, Johnson PJ. Galectin-1 on cervical epithelial cells is a receptor for the sexually transmitted human parasite *Trichomonas vaginalis*. Cell Microbiol. 2008;10(10):2078–2090. PMID: 18637021. doi:10.1111/j.1462-5822.2008.01190.x. 18637021 PMC4437540

[37] Kleshchenko YY, Moody TN, Furtak VA, Ochieng J, Lima MF, Villalta F. Human galectin-3 promotes *Trypanosoma cruzi* adhesion to human coronary artery smooth muscle cells. Infect Immun. 2004;72:6717–6721. PMID: 15501810. doi:10.1128/IAI.72.11.6717-6721.2004. 15501810 PMC523038

[38] Dings RPM, Miller MC, Griffin RJ, Mayo KH. Galectins as Molecular Targets for Therapeutic Intervention. Int J Mol Sci. 2018;19:905. PMID: 29562695. doi:10.3390/ijms19030905. PMC5877766 29562695

[39] Harvey KL, Jarocki VM, Charles IG, Djordjevic SP. The Diverse Functional Roles of Elongation Factor Tu (EF-Tu) in Microbial Pathogenesis. Front Microbiol. 2019;10:2351. PMID: 31708880. doi:10.3389/fmicb.2019.02351. 31708880 PMC6822514

[40] Zylicz M, Nieradko J, Taylor K. *Escherichia coli* dnaJ- and dnaK-gene products: synthesis in minicells and membrane-affinity. Biochem Biophys Res Commun. 1983;110:176–180. PMID: 6220698. doi:10.1016/0006-291x(83)91276-7. 6220698

[41] Bukau B, Reilly P, McCarty J, Walker GC. Immunogold localization of the DnaK heat shock protein in *Escherichia* coli cells. J Gen Microbiol. 1993;139:95–99. PMID: 8450312. doi:10.1099/00221287-139-1-95. 8450312

[42] Crapoulet N, Barbry P, Raoult D, Renesto P. Global transcriptome analysis of Tropheryma whipplei in response to temperature stresses. J Bacteriol. 2006;188(14):5228–5239. PMID: 16816195. doi:10.1128/JB.00507-06. 16816195 PMC1539978

[43] Trotta L, Weigt K, Schinnerling K, Geelhaar-Karsch A, Oelkers G, Biagi F, Corazza GR, Allers K, Schneider T, Erben U, et al. Peripheral T-Cell Reactivity to Heat Shock Protein 70 and Its Cofactor GrpE from *Tropheryma whipplei* Is Reduced in Patients with Classical Whipple’s Disease. Infect Immun. 2017;85:e00363–e00417. PMID: 28559404. doi:10.1128/IAI.00363-17. 28559404 PMC5520441

[44] Zhuo Y, Bellis SL. Emerging role of alpha2,6-sialic acid as a negative regulator of galectin binding and function. J Biol Chem. 2011;286:5935–5941. PMID: 21173156. doi:10.1074/jbc.R110.191429. 21173156 PMC3057866

[45] Levroney EL, Aguilar HC, Fulcher JA, Kohatsu L, Pace KE, Pang M, Gurney KB, Baum LG, Lee B. Novel innate immune functions for galectin-1: galectin-1 inhibits cell fusion by Nipah virus envelope glycoproteins and augments dendritic cell secretion of proinflammatory cytokines. J Immunol. 2005;175:413–420. PMID: 15972675. doi:10.4049/jimmunol.175.1.413. 15972675 PMC4428613

[46] Garner OB, Yun T, Pernet O, Aguilar HC, Park A, Bowden TA, Freiberg AN, Lee B, Baum LG, Doms RW. Timing of galectin-1 exposure differentially modulates Nipah virus entry and syncytium formation in endothelial cells. J Virol. 2015;89:2520–2529. PMID: 25505064. doi:10.1128/JVI.02435-14. 25505064 PMC4325760

[47] Ohshima S, Kuchen S, Seemayer CA, Kyburz D, Hirt A, Klinzing S, Michel BA, Gay RE, Liu FT, Gay S, et al. Galectin 3 and its binding protein in rheumatoid arthritis. Arthritis Rheum. 2003;48:2788–2795. PMID: 14558084. doi:10.1002/art.11287. 14558084

[48] Neidhart M, Zaucke F, von Knoch R, Jüngel A, Michel BA, Gay RE, Gay S. Galectin-3 is induced in rheumatoid arthritis synovial fibroblasts after adhesion to cartilage oligomeric matrix protein. Ann Rheum Dis. 2005;64:419–424. PMID: 15345499. doi:10.1136/ard.2004.023135. 15345499 PMC1755412

[49] Vilar KM, Pereira MC, Dantas AT, Rêgo MJBM, Pitta IDR, Marques CDL, Gonçalves RSG, Júnior LFDR, Duarte ÂLBP, Pitta MGDR. Galectin-1, -4, and -7 were associated with high activity of disease in patients with rheumatoid arthritis. Autoimmune Dis. 2019;2019:3081621. PMID: 31428469. doi:10.1155/2019/3081621. 31428469 PMC6681614

[50] Quartuccio L, Giovannini I, Pizzolitto S, Scarpa M, De Vita S. Seronegative arthritis and whipple disease: risk of misdiagnosis in the era of biologic agents. Case Rep Rheumatol. 2019;2019:3410468. PMID: 31737398. doi:10.1155/2019/3410468. 31737398 PMC6815603

[51] Glaser C, Rieg S, Wiech T, Scholz C, Endres D, Stich O, Hasselblatt P, Geißdörfer W, Bogdan C, Serr A, et al. Whipple’s disease mimicking rheumatoid arthritis can cause misdiagnosis and treatment failure. Orphanet J Rare Dis. 2017;12:99. PMID: 28545554. doi:10.1186/s13023-017-0630-4. 28545554 PMC5445468

[52] Seropian IM, González GE, Maller SM, Berrocal DH, Abbate A, Rabinovich GA. Galectin-1 as an Emerging Mediator of Cardiovascular Inflammation: mechanisms and Therapeutic Opportunities. Mediators Inflamm. 2018;2018:8696543. PMID: 30524200. doi:10.1155/2018/8696543. 30524200 PMC6247465

[53] Parsonage G, Trebilcock E, Toscano MA, Bianco GA, Ilarregui JM, Buckley CD, Rabinovich GA. Roles of galectins in chronic inflammatory microenvironments. Fut Rheumatol. 2006;1:441–454. doi:10.2217/17460816.1.4.441.

[54] Iwatani S, Shinzaki S, Amano T, Otake Y, Tani M, Yoshihara T, Tsujii Y, Hayashi Y, Inoue T, Okuzaki D, et al. Oligosaccharide-dependent anti-inflammatory role of galectin-1 for macrophages in ulcerative colitis. J Gastroenterol Hepatol. 2020;6:e1000722. Epub ahead of print. PMID: 32424849. doi:10.1111/jgh.15097. 32424849

[55] Chen SS, Sun LW, Brickner H, Sun PQ. Downregulating galectin-3 inhibits proinflammatory cytokine production by human monocyte-derived dendritic cells via RNA interference. Cell Immunol. 2015;294:44–53. PMID: 25684095. doi:10.1016/j.cellimm.2015.01.017. 25684095 PMC4704704

[56] Papaspyridonos M, McNeill E, de Bono JP, Smith A, Burnand KG, Channon KM, Greaves DR. Galectin-3 is an amplifier of inflammation in atherosclerotic plaque progression through macrophage activation and monocyte chemoattraction. Arterioscler Thromb Vasc Biol. 2008;28:433–440. PMID: 18096829. doi:10.1161/ATVBAHA.107.159160. 18096829

[57] Abebayehu D, Spence A, Boyan BD, Schwartz Z, Ryan JJ, McClure MJ. Galectin-1 promotes an M2 macrophage response to polydioxanone scaffolds. J Biomed Mater Res A. 2017;105:2562–2571. PMID: 28544348. doi:10.1002/jbm.a.36113. 28544348 PMC5563977

[58] Di Gregoli K, Somerville M, Bianco R, Thomas AC, Frankow A, Newby AC, George SJ, Jackson CL, Johnson JL. Galectin-3 Identifies a Subset of Macrophages With a Potential Beneficial Role in Atherosclerosis. Arterioscler Thromb Vasc Biol. 2020;40:1491–1509. PMID: 32295421. doi:10.1161/ATVBAHA.120.314252. 32295421 PMC7253188

[59] MacKinnon AC, Farnworth SL, Hodkinson PS, Henderson NC, Atkinson KM, Leffler H, Nilsson UJ, Haslett C, Forbes SJ, Sethi T. Regulation of alternative macrophage activation by galectin-3. J Immunol. 2008;180:2650–2658. PMID: 18250477. doi:10.4049/jimmunol.180.4.2650. 18250477

[60] Novak R, Dabelic S, Dumic J. Galectin-1 and galectin-3 expression profiles in classically and alternatively activated human macrophages. Biochim Biophys Acta. 2012;1820:1383–1390. PMID: 22155450. doi:10.1016/j.bbagen.2011.11.014. 22155450

[61] Yaseen H, Butenko S, Polishuk-Zotkin I, Schif-Zuck S, Pérez-Sáez JM, Rabinovich GA, Ariel A. Galectin-1 Facilitates Macrophage Reprogramming and Resolution of Inflammation Through IFN-β. Front Pharmacol. 2020;11:901. PMID: 32625094. doi:10.3389/fphar.2020.00901. 32625094 PMC7311768

[62] Lee PH, Liu CM, Ho TS, Tsai YC, Lin CC, Wang YF, Chen YL, Yu CK, Wang SM, Liu CC, et al. Enterovirus 71 virion-associated galectin-1 facilitates viral replication and stability. PLoS One. 2015;10:e0116278. PMID: 25706563. doi:10.1371/journal.pone.0116278. 25706563 PMC4338065

[63] Yang ML, Chen YH, Wang SW, Huang YJ, Leu CH, Yeh NC, Chu CY, Lin CC, Shieh GS, Chen YL, et al. Galectin-1 binds to influenza virus and ameliorates influenza virus pathogenesis. J Virol. 2011;85:10010–10020. PMID: 21795357. doi:10.1128/JVI.00301-11. 21795357 PMC3196456

[64] Caniglia JL, Guda MR, Asuthkar S, Tsung AJ, Velpula KK. A potential role for Galectin-3 inhibitors in the treatment of COVID-19. PeerJ. 2020;8:e9392. PMID: 32587806. doi:10.7717/peerj.9392. 32587806 PMC7301894

[65] Tzeng SF, Tsai CH, Chao TK, Chou YC, Yang YC, Tsai MH, Cha TL, Hsiao PW. O-Glycosylation-mediated signaling circuit drives metastatic castration-resistant prostate cancer. Faseb J. 2018;32:6869–6882. PMID: 29906246. doi:10.1096/fj.201800687. 29906246

[66] Kuo HY, Hsu HT, Chen YC, Chang YW, Liu FT, Wu CW. Galectin-3 modulates the EGFR signalling-mediated regulation of Sox2 expression via c-Myc in lung cancer. Glycobiology. 2016;26:155–165. PMID: 26447186. doi:10.1093/glycob/cwv088. 26447186

[67] Croci DO, Salatino M, Rubinstein N, Cerliani JP, Cavallin LE, Leung HJ, Ouyang J, Ilarregui JM, Toscano MA, Domaica CI, et al. Disrupting galectin-1 interactions with N-glycans suppresses hypoxia-driven angiogenesis and tumorigenesis in Kaposi’s sarcoma. J Exp Med. 2012;209:1985–2000. PMID: 23027923. doi:10.1084/jem.20111665. 23027923 PMC3478924

[68] Renesto P, Crapoulet N, Ogata H, La Scola B, Vestris G, Claverie JM, Raoult D. Genome-based design of a cell-free culture medium for *Tropheryma whipplei*. Lancet. 2003;362:447–449. PMID: 12927433. doi:10.1016/S0140-6736(03)14071-8. 12927433

[69] Trouplin V, Boucherit N, Gorvel L, Conti F, Mottola G, Ghigo E. Bone marrow-derived macrophage production. J Vis Exp. 2013;81:e50966. PMID: 24300014. doi:10.3791/50966. PMC3991821 24300014

[70] Fenollar F, Lagier JC, Raoult D. *Tropheryma whipplei* and Whipple’s disease. J Infect. 2014;69:103–112. PMID: 24877762. doi:10.1016/j.jinf.2014.05.008. 24877762

