# Supplementary material for: Human galectin-1 and galectin-3 promote Tropheryma whipplei infection
Source: Gut Microbes. 2021 Feb 12;13(1):1884515. doi: 10.1080/19490976.2021.1884515 (PMC7889132; doi:10.1080/19490976.2021.1884515)
Supplement: Supplemental Material [file KGMI_A_1884515_SM2327.zip › Supplementary information/GutMicrobes_Supplementary_DA.pdf]

Table S1. Summary of the proteins identified by MALDI-TOF mass spectrometry[a]

	Identification	Reference (NCBI/Uniprot)	Organism	Mass (Da)	coverage (%)	Score	Comment based on conserved domain search
Band_1	CCD domain-containing protein	Q83FH2_TROWT	Tropheryma whipplei (strain Twist)	225401	31	181	WiSP family membrane protein
	CCD domain-containing protein	Q83GX5_TROWT	Tropheryma whipplei (strain Twist)	243726	30	195	
Band_2	CCD domain-containing protein	Q83FH2_TROWT	Tropheryma whipplei (strain Twist)	225401	16	66	WiSP family membrane protein
	CCD domain-containing protein	Q83GX5_TROWT	Tropheryma whipplei (strain Twist)	243726	16	81	
Band_3	Uncharacterized protein	Q83GP3_TROWT	Tropheryma whipplei (strain Twist)	94775	20	54	WiSP family membrane protein
Band_4	WND domain-containing protein	Q83FU6_TROWT	Tropheryma whipplei (strain Twist)	88900	8	21	WiSP family membrane protein
Band_5	WND domain-containing protein	Q83FU6_TROWT	Tropheryma whipplei (strain Twist)	88900	11	39	WiSP family membrane protein
Band_6	Chaperone protein DnaK	DNAK_TROW8	Tropheryma whipplei (strain Twist)	65370	25	32	Chaperone protein DNAK
Band_7	Elongation factor Tu	EFTU_TROWT	Tropheryma whipplei (strain Twist)	43550	29	57	Elongation factor Tu
Band_8	Zinc-type alcohol dehydrogenase	Q83GG5_TROWT	Tropheryma whipplei (strain Twist)	37444	33	101	Zinc-type alcohol dehydrogenase
Band_9	Sugar ABC transporter substrate-binding protein (L-arabinose/D-xylose)	Q83MX9_TROWT	Tropheryma whipplei (strain Twist)	38929	40	75	Sugar ABC transporter substrate-binding protein (L-arabinose/D-xylose)

[a] Proteins were identified by peptide mass fingerprinting using Mascot software against available sequences in public database.

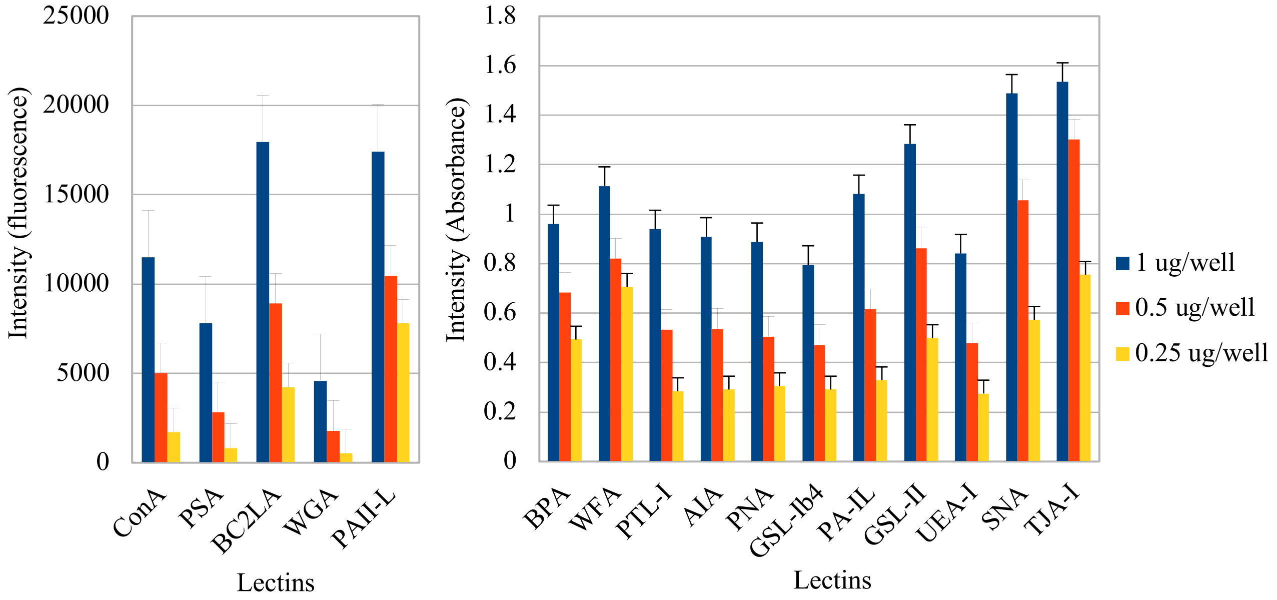

Supplementary figure 1. ***Lectin microarray for T. whipplei whole protein lysate.*** Presence of glucose, mannose, fucose, N-acetylglucosamine (GlcNAc), N-acetylgalactosamine (GalNAc), α-galactose, β-galactose and N-acetylneuraminic Acid (NeuAc)/ sialic acid in *T. whipplei* whole protein cell lysate, identified by lectin microarray performed using LEctPROFILE® plates for fluorescence (left) and absorbance (right). Experiment was performed on 3 different protein lysate, representative results of one experiment in duplicates are shown. Error bars represent the standard deviation of duplicates. *T. whipplei* whole protein lysate reactivity with different lectins indicated the presence of different glycans as follows: concanavalin A (ConA) for glucose and mannose, *Pisum sativum* agglutinin (PSA) for glucose, mannose and GlcNAc, *Burkholderia cenocepacia* lectin A (BC2LA) for mannose specific glycans, wheat germ agglutinin (WGA) for GlcNAc and NeuAc, *Pseudomonas aeruginosa* lectin (PA-IIL) for fucose, *Bauhinia purpurea* agglutinin (BPA), *Wisteria floribunda* lectin (WFL) and *Psophocarpus tetragonolobus* lectin I for GalNAc, *Artocarpus integrifolia* lectin (AIA) for galactose residues, peanut agglutinin for β-galactose, *Griffonia simplicifolia* lectin I isolectin B4 (GSL-Ib4) for α-galactose, *Pseudomonas aeruginosa* I

lectin (PA-IL) for galactose (Main target), fucose and mannose, *Griffonia simplicifolia* lectin II (GSL-II) for GlcNAc, *Ulex europaeus* agglutinin I (UEA-I) for fucose, *Trichosanthes japonica agglutinin I* (TJA-1) and *Sambucus nigra* agglutinin (SNA) for NeuAc.

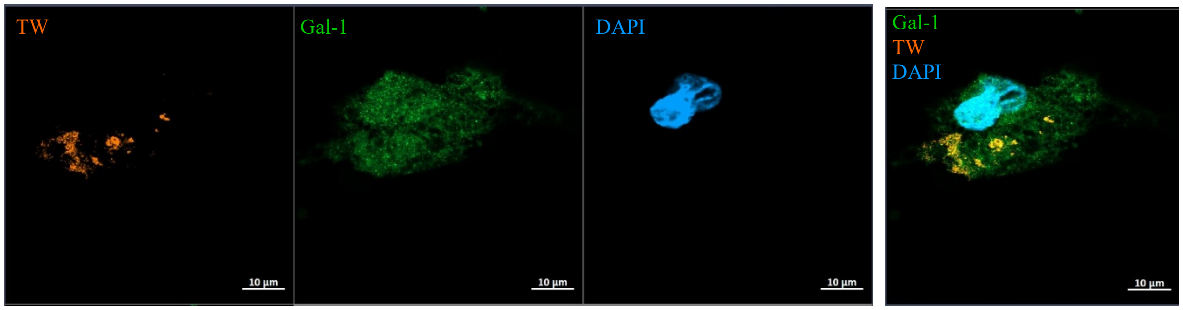

Supplementary figure S2. *Un-merged and merged immunofluorescence images of a macrophage infected with T. whipplei for 24 h.* *T. whipplei* (Orange) was detected with anti-*T. whipplei* antibody. Gal-1 (Green) was detected using rabbit anti-Gal-1 antibody and DNA was labelled with DAPI (blue).

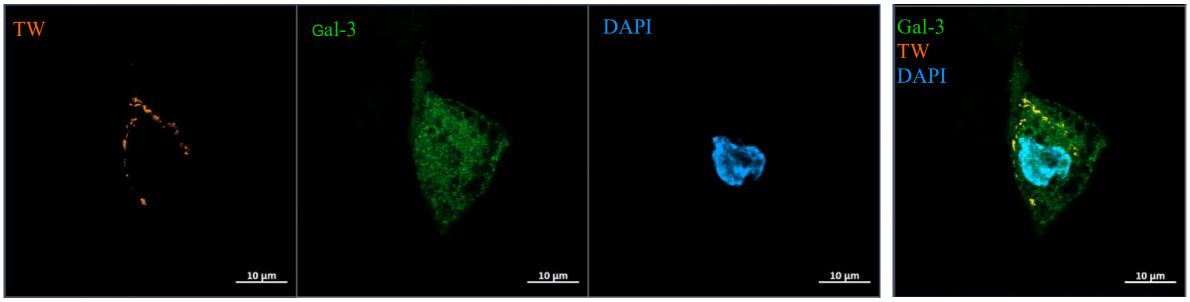

Supplementary figure S3. *Un-merged and merged immunofluorescence images of a macrophage infected with T. whipplei for 24 h.* *T. whipplei* (Orange) was detected with anti-*T. whipplei* antibody. Gal-3 (Green) was detected using rabbit anti-Gal-3 antibody and DNA was labeled with DAPI (blue).

Supplementary experimental procedures

Lectin microarray

GLYcoPROFILE® of *T. whipplei* lysates were performed according to the GLYcoDiag's protocol already described (Landemarre and Duverger, 2013) and carried out with the customized LEctPROFILE® plates (96-wells plates for fluorescence and absorbance) obtained from GLYcoDiag (Orléans, France). Briefly, *T. whipplei* whole cell protein lysates were first labeled with biotin. Then, three different concentrations as 1 µg/well, 0.5 µg/well and 0.25 µg/well from biotin-labeled protein lysate were deposited into plate wells and incubated for 2 h at room temperature. Plates were then washed with PBS and incubated for 30 min with the conjugate (Extravidine-peroxidase for absorbance or streptavidine-DTAF for fluorescence) and washed again with PBS. For absorbance plates, 100 µl from OPD (SIGMAFAST™ OPD (o-Phenylenediamine dihydrochloride) solution was added and incubated again for 15 min to detect peroxidase activity. Coloration was stopped by adding 100 µl of 1 mM HCl. The readout is performed with an absorbance reader (Fluostar, BMG labtech, Offenburg, Germany). For fluorescence plate, 100 µl of PBS was added before the readout.

Supplemental References

Landemarre L, Duverger E. Lectin glycoprofiling of recombinant therapeutic interleukin-7. Methods Mol Biol. 2013; 988:221-6. doi: 10.1007/978-1-62703-327-5_14. PMID: 23475723.
